# Additional Nitrogen Fertilization at Heading Time of Rice Down-Regulates Cellulose Synthesis in Seed Endosperm

**DOI:** 10.1371/journal.pone.0098738

**Published:** 2014-06-06

**Authors:** Keiko Midorikawa, Masaharu Kuroda, Kaede Terauchi, Masako Hoshi, Sachiko Ikenaga, Yoshiro Ishimaru, Keiko Abe, Tomiko Asakura

**Affiliations:** 1 Department of Applied Biological Chemistry, Graduate School of Agricultural and Life Sciences, The University of Tokyo, Bunkyo-ku, Tokyo, Japan; 2 Crop Development Division, NARO Agricultural Research Center, Inada, Joetsu, Niigata, Japan; 3 Field Crop and Horticulture Research Division, NARO Tohoku Agricultural Research Center, Morioka, Iwate, Japan; 4 Food Safety and Reliability Project, Kanagawa Academy of Science and Technology, Takatsu-ku, Kawasaki, Kanagawa, Japan; ISA, Portugal

## Abstract

The balance between carbon and nitrogen is a key determinant of seed storage components, and thus, is of great importance to rice and other seed-based food crops. To clarify the influence of the rhizosphere carbon/nitrogen balance during the maturation stage of several seed components, transcriptome analysis was performed on the seeds from rice plants that were provided additional nitrogen fertilization at heading time. As a result, it was assessed that genes associated with molecular processes such as photosynthesis, trehalose metabolism, carbon fixation, amino acid metabolism, and cell wall metabolism were differentially expressed. Moreover, cellulose and sucrose synthases, which are involved in cellulose synthesis, were down-regulated. Therefore, we compared cellulose content of mature seeds that were treated with additional nitrogen fertilization with those from control plants using calcofluor staining. In these experiments, cellulose content in endosperm from plants receiving additional nitrogen fertilization was less than that in control endosperm. Other starch synthesis-related genes such as starch synthase 1, starch phosphorylase 2, and branching enzyme 3 were also down-regulated, whereas some α-amylase and β-amylase genes were up-regulated. On the other hand, mRNA expression of amino acid biosynthesis-related molecules was up-regulated. Moreover, additional nitrogen fertilization caused accumulation of storage proteins and up-regulated Cys-poor prolamin mRNA expression. These data suggest that additional nitrogen fertilization at heading time changes the expression of some storage substance-related genes and reduces cellulose levels in endosperm.

## Introduction

Regulation of carbon (C) and nitrogen (N) metabolism is indispensable for plant growth and development. Carbon and nitrogen species are essential constituents of both macronutrients and signaling metabolites, which influence several cellular processes and gene expression [1]–[6]. In crop plants, starch and protein content in seed are determinants of yield and quality. These are synthesized using sugars and amino acids from the plant body, and share photosynthetic carbon sources for their synthesis. Thus, control of C/N balance during the reproductive stage is critical for high-yield and high-quality crop production.

The amount and timing of nitrogen fertilization are the most important factors for beneficial control of C/N balance, because the distribution of carbon sources from photosynthesis is generally influenced by plant nitrogen conditions [7], [8]. For example, the expression of photosynthetic and carbon fixation-related genes rapidly decreases in rice roots and leaves under low nitrogen conditions [9]. Under these conditions, rice leaves turn to pale green, carbon fixation is reduced, and remobilized nitrogen is used for other metabolic processes. In contrast, additional nitrogen fertilization facilitates maturation and elevates grain yields in commercial rice cultivation [10]. A high-nitrogen condition retards leaf senescence by maintaining nitrogen-containing compounds such as chlorophyll and photosynthetic proteins, thus elevating photosynthetic activity and transport of photosynthetic materials to the seed throughout the seed-maturation period. Such conditions may alter accumulation of seed components. In fact, protein content of rice seeds is elevated under conditions of high nitrogen fertilization [11].

Above results suggest that rhizospheric nitrogen influences global gene expression in the plant body, thereby implying the existence of gene networks that control C/N balance. In addition, gene expression in seeds is strongly influenced by the condition of the plant body. However, no studies have examined the influence of nitrogen on gene expression, metabolic processes, and accumulation of components in rice seeds. In this study, we examined the effects of nitrogen fertilization at heading time of rice, because plant body sizes and numbers of spikelets are fixed before fertilization. Rhizospheric nitrogen may directly affect metabolic processes during seed maturation. Thus, we examined changes in gene expression that correspond with nitrogen fertilization at heading time using DNA microarray analysis. Subsequently, we examined whether these changes in gene expression are correlated with seed components. The present data indicate that nitrogen fertilization at heading time decreases cellulose synthesis.

## Materials and Methods

### Rice Cultivation under Field Conditions

*Oryza sativa* L. cv. Nipponbare was used in all experiments. Rice plants were grown in a paddy field at the NARO Agricultural Research Center, Niigata, Japan. Field trials were conducted for three years.

At heading time, the experimental field was divided into a control plot and a nitrogen-fertilized (N-fertilized) plot using a plastic board. Ammonium chloride was sprayed on the soil surface of the N-fertilized section at a rate of 8 kg/1,000 m^2^. The sampling area contained 10×10 plants in each section, and all mature grains in the sampling area were harvested and prepared for analysis of nitrogen and amino acid content.

### Rice Cultivation in a Plant Incubator

To improve reproducibility, a plant incubator with fluorescent lamps on inner walls (model FLI2000A, Tokyo Rikakikai, Tokyo, Japan) was used to simulate paddy field conditions.

**Figure 1:**
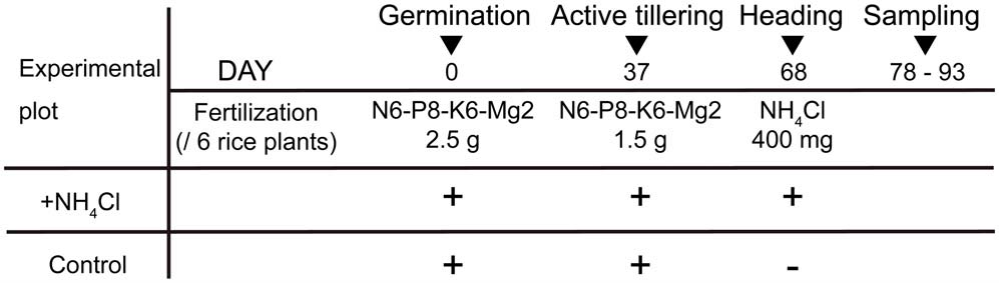
Rice cultivation schedule. Samples were grown in a growth chamber at 28°C/22°C over a 12-h light/12-h dark cycle. Fertilizer was supplied at planting and 37 days after germination. In addition, 400 mg ammonium chloride (NH_4_Cl) was supplied at heading time to the “+NH_4_Cl” group.

The schedule of cultivation is shown in Fig. 1. Plastic containers (C-AP fruit 500-1; 173×123×70 mm; Chuo Kagaku, Saitama, Japan) were filled with 500 mL rice nursery soil (Honen Agri, Niigata, Japan) and were supplied with 2.5 g fertilizer containing 0.15 g nitrogen, 0.2 g phosphate, 0.15 g potassium, and 0.05 g magnesium. Six plants were cultivated in each plastic container. Each of the four containers was placed on stainless trays (TRAY SUS No. 9, 367×257×92 mm; AZ ONE, Osaka, Japan); two trays, one for the control plot and the other for the N-fertilized plot, were placed in an incubator. Each plot contained 24 plants and tap water was provided to the depth such that the container was submerged. These plants were cultivated under short-daytime conditions with a 12-h maximum illumination (28°C)/12-h dark (22°C) cycle. Water depth was recovered every 2 days using tap water. The growth of each plant was restricted to the main culm by removing tillers. After 5 weeks, 1.5 g fertilizer was supplied to each container. At heading stage, ammonium chloride was sprayed on the soil surface of the N-fertilized plot tray at a rate of 400 mg/container.

To examine the synthesis of storage materials in more detail, samples were taken at 10, 15, 20, and 25 DAF during incubator cultivation. Spikelets were marked as for field cultivation and were subsequently sampled. Developing grains were collected randomly from 24 plants in each experimental plot. All dehusked grains from the same sampling time were mixed, frozen in liquid nitrogen, and stored at −80°C until use. For microarray analysis, RNAs from 15-DAF grains were used, because storage materials are synthesized actively during this stage [12]. Finally, mature grains were harvested from each plant and their nitrogen content was determined.

### Analysis of Nitrogen Content, Amino Acid Composition, and Protein Composition

Nitrogen content was determined using the Kjeldahl method in field trials. Amino acid analysis was performed at the Japan Food Research Laboratories (http://www.jfrl.or.jp/e/index.html; Tokyo, Japan) using an automated amino acid analyzer and HPLC according to their standard protocol. Total amino acid content was analyzed after hydrolysis of rice samples using hydrogen chloride. Free amino acid content was analyzed using sulfosalicylic acid- extractable fraction of rice samples. After harvest of all three trials, polished rice seeds were ground to powder in a cyclone sample mill (Shizuoka Seiki, Shizuoka, Japan), and nitrogen and amino acid content were determined for each trial. The sum of individual amino acid content was calculated, and differences between means were used for statistical analysis.

Nitrogen content was determined using a rapid-N-III automated nitrogen analyzer (Elementar Analysensysteme GmbH, Hanau, Germany) in incubator cultivation. Three independent plants from each fertilization division were selected, and nitrogen content of six polished grains from each plant was determined.

Protein composition of polished rice was analyzed using SDS-PAGE as shown previously with some modifications [13]. Subsequently, six seeds from each plant were ground to a powder, and soluble protein was extracted by adding 2.4 mL globulin extraction buffer containing 10 mM Tris-HCl (pH 7.5), 1 mM EDTA, and 0.5 M NaCl. Insoluble protein was then extracted by adding 2.4 mL of the total protein extraction buffer containing 50 mM Tris-HCl, (pH 6.8), 8 M urea, 4% SDS, and 5% 2-mercaptoethanol. Subsequently, a 10 µL aliquot of extracted protein was used for SDS-PAGE.

### Total RNA Isolation and DNA Microarray Preparation

Seeds at 15 DAF were collected from 24 control plants and 24 N-fertilized plants, immediately frozen in liquid nitrogen and pooled. Three sets of five seeds were selected from each seed pool. Samples were then ground to a fine powder, and RNA was extracted using an RNeasy Plant Mini kit (Qiagen, Hilden, Germany).

DNA microarray analysis was performed using a One-Cycle cDNA Synthesis kit (Affymetrix, Santa Clara, CA), a Sample Cleanup Module (Affymetrix), and a GeneChip IVT Labeling kit (Affymetrix). Experimental procedures were performed according to the manufacturer’s protocols. Briefly, after synthesizing cDNA from purified RNA, biotinylated cRNA was transcribed from cDNA using T7 RNA polymerase, fragmented, and was then added to a GeneChip Rice Genome Array containing over 50,000 rice genes (Affymetrix). Fragmented RNA was hybridized with the array at 45°C for 16 h. The array was then washed, labeled with phycoerythrin, and scanned for fluorescence using the Affymetrix GeneChip System. All microarray data were submitted to the National Center for Biotechnology Information (NCBI) Gene Expression Omnibus database (http://www.ncbi.nlm.nih.gov/geo/; GEO Series ID GSE49818). Further, Affymetrix GCOS software was used to convert array images and probe intensities to CEL files. CEL files were then transferred to a personal computer and were analyzed using R statistical language and Bioconductor software. Files were normalized using the distribution-free weighted (DFW) method [14]. The pvclust function was then used to perform clustering analysis of the samples.

Gene expression patterns in rice grown under +NH_4_/−NH_4_ conditions were compared using the “rank products” function [15]. Genes with false discovery rates (FDR) of <0.05 were extracted, analyzed with the “pvclust” function [16], and divided according to their expression patterns. Identified genes were analyzed using the annotation file for the Rice Genome Array, which was downloaded from the NetAffx database on the Affymetrix website.

Gene-annotation enrichment analysis of differentially expressed genes (DEGs) was performed using the Database for Annotation, Visualization and Integrated Discovery (http://david.abcc.ncifcrf.gov/) [17] and Quick GO (http://www.ebi.ac.uk/QuickGO/) [18]. EASE scores from modified Fisher’s exact test *P*-values [19] were used to extract statistically over represented GO terms from DEGs. When annotations could not be obtained using the NetAffx database, we consulted the following databases: Rice TOGO (http://agri-trait.dna.affrc.go.jp/index.html), RiceXPro (http://agri-trait.dna.affrc.go.jp/index.html), MSU Rice Genome Annotation Project (http://rice.plantbiology.msu.edu/), RAP-DB (http://rapdblegacy.dna.affrc.go.jp/), and UniProt (http://www.uniprot.org/).

### β-glucan Staining

β-glucan staining was performed on 30 mature seeds that were randomly selected from each fertilization plot in the third field trial. Fresh-frozen rice grain sections were prepared for fluorescence microscopy as described in Saito *et al.*, (2008). Frozen sections (5 µm thick) were cut from each seed, soaked in a 10% (w/v) potassium hydroxide solution, and were then stained with 0.05% (w/v) calcofluor white for 1–2 min. Further, sections were observed under a microscope using UV illumination. ImageJ software (http://rsbweb.nih.gov/ij/) was used to quantify fluorescence intensities. Fluorescence intensity per unit area in the part of endosperm that was surrounded by the dotted lines was measured was adjusted by subtraction of background fluorescence intensity.

## Results and Discussion

In this study, the timing of additional nitrogen fertilization is the key factor. At heading time, plant body growth ceases and number of spikelets are fixed. Therefore, the effects of additional nitrogen (ammonium) are likely to be reflected in seed maturation and storage material synthesis.

**Figure 2:**
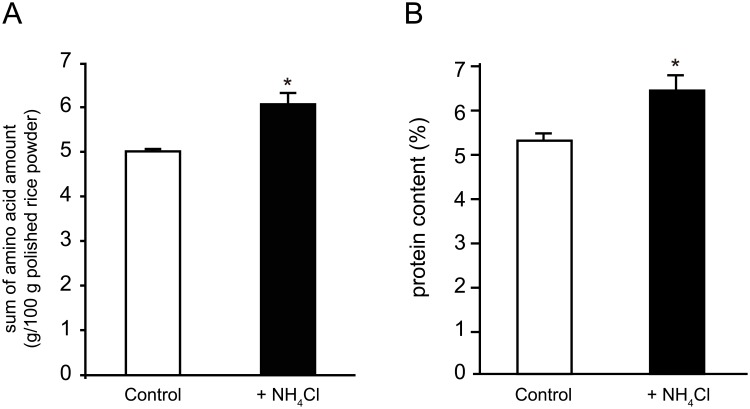
Amino acid and protein contents of mature polished rice cultivated in a field. Field trials were conducted for three years and data are expressed as means of the three trials. Filled bars correspond with samples from the N-fertilized plot and unfilled bars correspond with samples from the control plot. Error bars represent standard deviation (SD; *P<0.01) (A) Total amino acid content was analyzed after hydrolysis using hydrogen chloride. (B) Protein content was estimated using the Kjeldahl method.

In general, enriched nitrogen fertilization elevates protein content, as determined using the Kjeldahl method [10]. In agreement, the sum of amino acid and protein content, were elevated in mature rice treated with additional fertilization in all trials (Fig. 2 and Table S1). As shown in Table S1, amino acid content was also higher in individuals from the N-fertilized plot in all trials. However, free amino acid levels remained low or undetectable in all trials (Table S1). These results indicate that nitrogen fertilization at heading time was mainly utilized for protein synthesis and not for the accumulation of free amino acids. At the third field trial, we took some preliminary results about developing grains. Grain weight did not differ significantly between plants from the N-fertilized and control plot at every sampling time (data not shown). Although differences were not statistically confirmed, seed nitrogen content was higher in the N-fertilized plot than that in the control plot at every sampling time. It is a reasonable speculation that gene expression in developing grain also changes in response to additional nitrogen fertilization.

**Figure 3:**
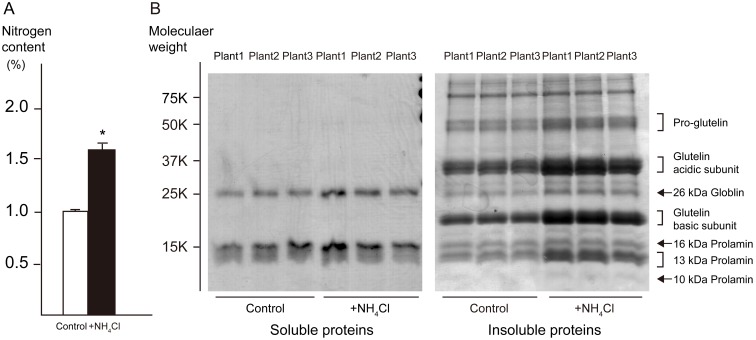
Analysis of proteins in polished rice from incubator cultivated plants. Three independent plants were selected from the N-fertilized and control plots. (A) Nitrogen content of polished rice was determined using a Rapid-N analyzer. Error bars represent standard deviation (SD; *P<0.001) (B) Protein composition of polished rice was analyzed using SDS-PAGE. Soluble proteins and insoluble proteins were extracted sequentially from the same sample. Major storage proteins in rice seed were extracted as insoluble proteins, as indicated at the right side of the gel.

To investigate the genomic response to nitrogen fertilization, a plant incubator was used to control growth conditions between replicate experiments. Rice plants from the two experimental plots were morphologically similar at heading stage, although the leaf blades of N-fertilized rice clearly showed a darker green color than those of the control group (Fig. S1). Mature seeds were obtained from all but one control plant. Moreover, the average maturation rate was calculated from the number of mature seeds divided by the number of spikelets, and was found to be 88.0% in the N-fertilization plot and 92.2% in the control plot. Nitrogen content of mature polished grains was much higher in plants from the N-fertilized plot (Fig. 3A), and this difference of seed nitrogen content between two plots was greater in incubator cultivation than in field trial. In incubator cultivation, water was pooled in trays and plant sizes were reduced by removing tillers. Thus, all fertilizer remained in incubator trays, allowing for higher quantities of nitrogen to be utilized for grain development. Accordingly, higher nitrogen content of seeds from the N-fertilized plot was reflected by band density of insoluble proteins in SDS-PAGE analysis (Fig. 3B). Each density corresponding to glutelin and a 13 kDa prolamin was higher in the case of N-fertilized plot.

**Figure 4:**
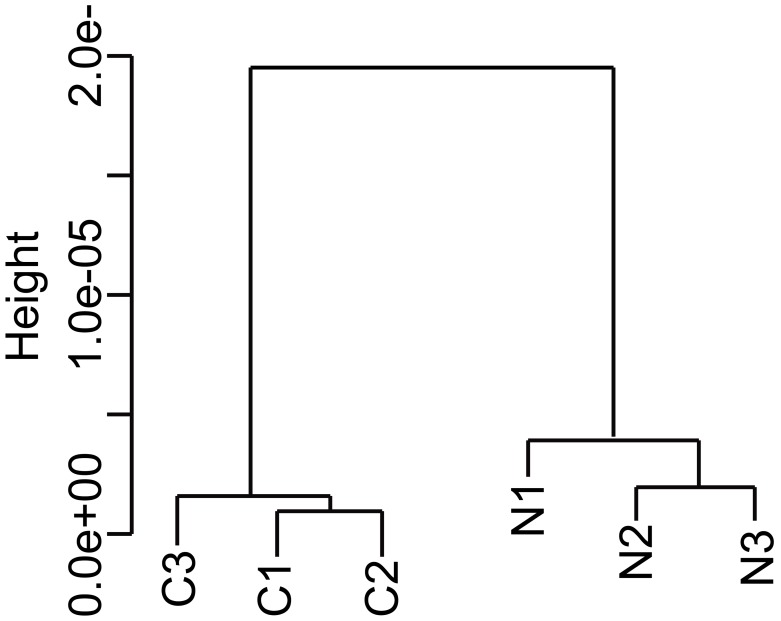
Cluster dendrogram. A cluster dendrogram was generated using rice seed gene expression data from six samples and the “pvclust” function. Each sample was prepared at 15-DAF seeds from 24 control or 24 N-fertilized plants and were pooled. Three sets of five seeds were picked from each seed pool. C, control samples; N, N-fertilized samples.

Dehusked 15-DAF developing seeds from rice plants were subjected to DNA microarray analysis, and data from six samples (three per experimental plot) were normalized using the DFW method and subjected to hierarchical clustering analysis. As shown in Fig. 4, a distinct separation was observed between the N-fertilized and control samples.

**Figure 5:**
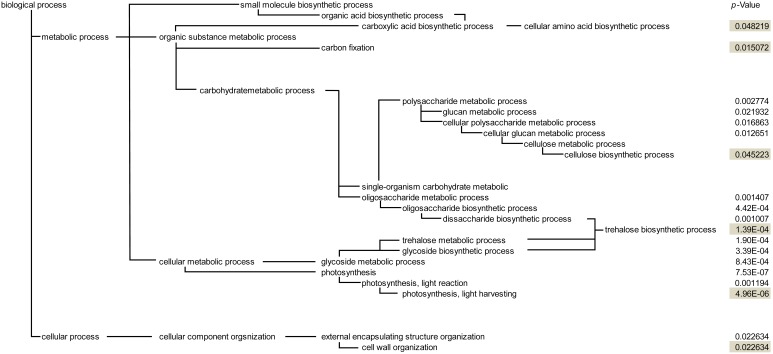
Significantly enriched categories were identified using QuickGO. In response to additional fertilization, 1,365 genes were up- or down-regulated; FDR-corrected *P*-values of categories at the deepest hierarchical level are shaded; *P<0.05.

Furthermore, DEGs were identified between the control and N-fertilized samples using the rank products method. DEGs with FDR<0.05 were extracted, revealing 678 significantly up-regulated genes and 687 significantly down-regulated genes due to additional fertilization (Tables S2 and S3). The extracted DEGs were classified into functional categories according to gene ontology (GO) terms. Significantly enriched categories of DEGs (FDR<0.05) are shown in Fig. 5. The hierarchical GO structure indicated a more specific category with a deeper hierarchy. Therefore, most important categories appeared at the lower end of the tree (depth of hierarchy is shown in shadowed areas; Fig. 5). Furthermore, six functional categories were significantly enriched in DEGs. These included “photosynthesis, light harvesting,” “trehalose biosynthetic process,” “carbon fixation,” “cell wall organization,” “cellulose biosynthetic process,” and “cellular amino acid biosynthetic process.” Genes that were predicted to be involved in the synthesis of storage substances were also identified from the list of DEGs (Table 1). The “photosynthesis, light harvesting” category included genes that encode chlorophyll-binding proteins, and Rubisco was identified from the “carbon fixation” category (Table 2). Consistent with these results, the leaf blades of the N-fertilized samples were clearly darker in color than those of the control blades (Fig. S1). Because DNA microarray samples were collected from 15-DAF seeds with green pericarps, the molecules mentioned above are likely localized to the pericarp.

**Table 1:** Genes related to synthesis of storage substances in seeds with altered mRNA expression in response to additional fertilizer.

Probe set ID	RAP-DB ID	RAP-DB Annotation	Gene Expression
Os.26109.1.S1_x_at	Os05g0329100, Os05g0329200, Os05g0329400, Os05g0330600	Cysteine-poor 13 kDa prolamin	Up
Os.25998.1.S1_at	Os05g0329100, Os05g0329200, Os05g0329400, Os05g0330600	Cysteine-poor 13 kDa prolamin	Up
Os.8502.4.S1_at	Os05g0329300	Cysteine-poor 13 kDa prolamin	Up
Os.8502.5.S1_x_at	Os05g0328333, Os05g0329100, Os05g0329200, Os05g0329400, Os05g0330600, Os07g0219300, Os07g0219400, Os07g0220050	Cysteine-poor 13 kDa prolamin	Up
Os.20396.1.A1_at	Os02g0456150	11-S plant seed storage protein family protein.	Up
Os.20396.1.A1_s_at	Os02g0456150	11-S plant seed storage protein family protein.	Up
OsAffx.2749.1.S1_at	Os02g0456150	11-S plant seed storage protein family protein.	Up
Os.17979.1.S1_at	Os02g0244100	Grain weight 2 (OsGW2).	Up
OsAffx.16823.1.S1_at	Os08g0137250	Fertilization-Independent endosperm(Protein Fertilization-Independent Seed 3) (OsFIE1).	Up
Os.12593.1.S1_s_at	Os08g0473600	Alpha-amylase isozyme 3E precursor (EC 3.2.1.1).	Up
Os.10339.1.S1_at	Os03g0141200	Similar to Beta-amylase PCT-BMYI (EC 3.2.1.2).	Up
Os.46618.1.S1_at	Os10g0565200	Similar to Beta-amylase PCT-BMYI (EC 3.2.1.2).	Up
Os.13907.1.S1_at	Os02g0248800	Similar to Glutelin type-B 2 precursor.	Up
Os.29800.1.S1_x_at	Os01g0702900, Os02g0771500	Sucrose-phosphate synthase (EC 2.4.1.14).	Up
Os.12725.1.S1_at	Os06g0160700	Similar to Starch synthase I, chloroplast precursor (EC 2.4.1.21)(Soluble starch synthase 1) (SSS 1).	Down
Os.2623.1.S1_at	Os01g0851700	Similar to Cytosolic starch phosphorylase (Fragment)/Starch phosphorylase 2.	Down
Os.4179.1.S1_at	Os02g0528200	Branching enzyme-3 precursor (EC 2.4.1.18).	Down
OsAffx.13550.1.S1_s_at	Os03g0808200	UDP-glucuronosyl/UDP-glucosyltransferase family protein.	Down
Os.33722.1.S1_at	Os01g0736100	UDP-glucuronosyl/UDP-glucosyltransferase family protein.	Down
Os.9127.1.S1_a_at	Os06g0194900	Sucrose synthase 2 (EC 2.4.1.13).	Down
Os.9860.1.S1_at	Os07g0616800	Sucrose synthase 3 (EC 2.4.1.13).	Down

**Table 2:** Differentially expressed genes in significantly enriched GO categories (*P<0.05).

	RAP-DB ID	RAP-DB annotation	Gene Expression
Photosynthesis, light harvesting	Os08g0435900, Os07g0562700, Os07g0558400, Os01g0600900, Os03g0592500, Os04g0457000	Chlorophyll a/b-binding protein	Up
Trehalose biosynthetic process	Os03g0386500	Trehalose-6-phosphate phosphatase 9	Up
	Os02g0661100	Trehalose-6-phosphate phosphatase 1	Down
	Os09g0369400	Trehalose-6-phosphate phosphatase 7	Down
	Os01g0730300	Trehalose-6-phosphate synthase 3	Down
	Os02g0790500	Trehalose-6-phosphate synthase 5	Up
	Os05g0517200	Trehalose-6-phosphate synthase 6	Up
Carbon fixation	Os01g0791033, Os05g0427800, Os12g0207600	Ribulose bisphosphate carboxylase large chain precursor (EC 4.1.1.39) (RuBisCO large subunit).	Up
	Os12g0292400, Os12g0291400	Ribulose bisphosphate carboxylase small chain	Up
Cell wall organization	Os10g0555900, Os10g0548600	Beta-expansin precursor	Up
	Os08g0160500	Cellulose synthase-like protein F6 (OsCslF6)	Down
	Os10g0450900	Glycine-rich cell wall structural protein 2 precursor	Up
	Os07g0208500	Cellulose synthase A8 (OsCESA8)	Down
	Os02g0130200	Virulence factor, pectin lyase fold family protein	Up
	Os07g0252400	Cellulose synthase A6 (OsCESA6)	Down
	Os02g0738600	Endoglucanase 7	Down
	Os03g0377700	Cellulose synthase-like A5 (CSLA5)	Down
	Os03g0808100	Cellulose synthase A2 (OsCESA2)	Down
	Os03g0837100	Cellulose synthase A5 (OsCESA5)	Down
	Os08g0237000	Xyloglucan endotransglycosylase/hydrolase protein 8 precursor(End-xyloglucan transferase) (OsXTH8)	Down
Cellulose biosynthetic process	Os07g0252400	Cellulose synthase A6 (OsCESA6)	Down
	Os08g0160500	Cellulose synthase-like protein F6 (OsCslF6)	Down
	Os07g0208500	Cellulose synthase A8 (OsCESA8)	Down
	Os03g0808100	Cellulose synthase A2 (OsCESA2)	Down
	Os03g0837100	Cellulose synthase A5 (OsCESA5)	Down
Cellular amino acid biosynthetic process	Os04g0669800	Methylthioribose kinase	Up
	Os01g0720700	Serine acetyltransferase 1	Up
	Os11g0256000	Acetolactate synthase, small subunit family protein	Up
	Os09g0565700	Prephenate dehydratase domain containing protein	Up
	Os12g0578200	Chorismate mutase, chloroplast precursor (CM-1)	Up
	Os03g0291500	Asparagine synthase domain containing protein	Up
	Os01g0681900	NADH - Glutamate Synthase 1	Down
	Os03g0279400	Arginine biosynthesis bifunctional protein ArgJ, chloroplastic	Down
	Os02g0510200	Acetohydroxyacid synthase	Up
	Os03g0826500	Anthranilate synthase alpha 1 subunit	Up
	Os03g0389700	Phospho-2-dehydro-3-deoxyheptonate aldolase 1, chloroplastic	Up

Only annotated genes are listed. All 1365 differentially expressed genes are listed in Tables S2 and S3.

**Figure 6:**
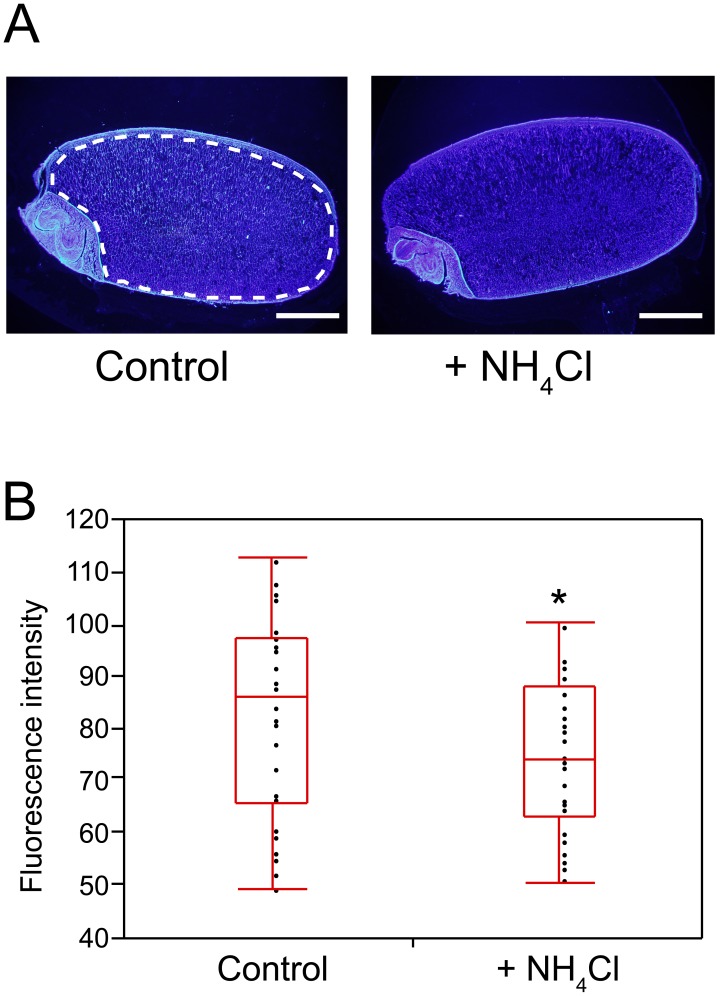
β-Glucan content of rice grains. (A) Histochemical staining of mature rice seed. Cross-sections of grains from the control plot (left) and from the N-fertilized plot (+NH_4_Cl; right) are shown. Sections were stained for β-glucan using calcofluor white. The white-dotted line indicates the area analyzed for fluorescence intensity. The fluorescence intensity of the endosperm was calculated by adjusting the background intensity. Scale bars, 1 mm. (B) Endosperm fluorescence intensity was analyzed using ImageJ. Sections were cut from 30 randomly selected grains from each plot. The horizontal line inside the box plot indicates the median value. The inner box indicates the interquartile range and runs between 25^th^ and 75^th^ percentiles. The upper line extending from the box indicates the largest value between the 75^th^ percentile and the point at 1.5 times the interquartile range. The lower line extending from the box indicates the smallest point between the 25^th^ percentile and the point at 1.5 times the interquartile range; *P<0.05.

Cell wall-related genes such as those encoding cellulose synthase A catalytic subunit (CESA) and xyloglucan endotransglucosylase/hydrolase, were down-regulated following additional fertilization (Table 2). Genes encoding OsCESA2, OsCESA5, OsCESA6, OsCESA8, CESA-like protein A5 (*OsCslA5*), and CESA-like protein F6 (*OsCslF6*) are all involved in cell wall synthesis, and were down-regulated in the presence of additional fertilization. Thus, seeds were examined to determine whether β-glucan constituents of cellulose and (1, 3; 1, 4)-β-D-glucan actually decreased. Staining with calcofluor white demonstrated weaker fluorescence intensity in seed endosperms from the N-fertilized plot than from the control plot (Fig. 6). Cell walls of rice endosperm comprise cellulose and hemicellulose [21]. Rice hemicellulose comprises arabinoxylans and (1, 3; 1, 4)-β-D-glucan, also known as mixed-linkage glucan (MLG). Although calcofluor white stains both cellulose and MLG, rice does not accumulate significant amount of MLG in its grains [21], [22]. Thus the present data suggest decreased cellulose content of endosperm. In general, cellulose is synthesized from UDP-glucose by CESA, which comprises a cellulose synthase complex of six subunits [23]–[25]. Although at least 11 genes have been annotated as CESA in the MSU rice genome annotation project, the functions of only three genes, *OsCESA4*, *OsCESA7*, and *OsCESA9*, have been examined [26]. Sucrose synthase (SUS), which is involved in UDP-glucose metabolism, was down-regulated by additional fertilization, and plays a direct role in cell wall biosynthesis by forming a complex with CESA [27]–[30]. Six SUS homologs are present in the rice genome [31]. Among these, *SUS2* and *SUS3* were extracted from DEGs as genes that were down-regulated by additional fertilization (Table 1). In particular, SUS3 is reportedly localized primarily in the endosperm and in the aleurone layer [31], [32], indicating that CESA2, CESA5, CESA6, and CESA8 may form a complex with SUS3 to synthesize cellulose in the endosperm. The relationship between rice quality and cellulose content remains poorly understood. However, it is accepted that rice with a high nitrogen content has less stickiness, greater hardness after cooking, less palatability, and less processing quality [33]–[35]. Accordingly, cell walls may become harder with increased cellulose content. However, in the present experiments, rice with high nitrogen content contained less cellulose. Thus, the complexities of relationship between cooked-rice properties and rice-seed components require further detailed study.

Other polysaccharide-related genes were identified from DEGs with FDR<0.05 (Table 1). The genes encoding starch synthase 1, starch branching enzyme 3, and starch phosphorylase 2 were down-regulated. These enzymes participate in starch synthesis from UDP-glucose. However, α-amylase and β-amylase, which further process starch into smaller sugars, were up-regulated in plants receiving additional fertilization (Table 1). Although various α-amylases are present in rice, only *RAmy3E* (Os08g0473600) was identified in this study, and is reportedly localized in seeds [36]–[38]. Because genes associated with starch synthesis were down-regulated concomitantly with increases in mRNA expression of starch-degrading enzymes, the starch content of rice may have decreased. This phenomenon was previously reported in rice kept under high temperature conditions during maturation [39]. Besides, starch synthesis is reportedly suppressed when plant bodies are subjected to carbon starvation, favoring monosaccharide production [36], [40].

Some genes of cellular amino acid biosynthetic processes, expect for NADH-glutamate synthase 1 and arginine biosynthesis bifunctional protein, were up-regulated, (Table 2 and Fig. S2). This result indicates that amino acid metabolism was activated to meet the demands of storage protein synthesis. In fact, amino acid and storage protein content were increased in polished rice from the N-fertilized plot (Figs. 2 and 3). Also, DNA microarray analysis showed remarkable increase in mRNA expression of storage proteins, in particular that of Cys-poor prolamins. Glutelin B2 and Os02g0456150 mRNA, annotated as “11 S-plant seed storage protein family protein”, were also increased. The latter gene was found to be a homologous pseudogene of glutelin C. Other storage proteins were not extracted in our analysis, suggesting that mRNA response to additional nitrogen fertilization varies between types of storage protein.

Prolamin comprises several molecular species of various sizes, including 10-kDa, Cys-poor 13-kDa, Cys-rich 13-kDa, and 16-kDa prolamins [20], [41], [42]. Cys-poor prolamins are accumulated at late stages of grain filling [43], and their expression is increased during suppression of glutelins, which are the most abundant storage protein in rice [44]. Both the present data and previous reports suggest that the expression of Cys-poor prolamin is controlled at the mRNA level, and effectively stores excess nitrogen during additional fertilization [43], [44]. This may be because Cys-poor prolamin has the simple primary structure with the absence of cysteine residues, and is directly accumulated in the endoplasmic reticulum where protein synthesis occurs. Rice with a high protein content shows poor cooking quality, and prolamins are suggested to associate such phenomena [33]–[35], [45]. Thus, Cys-poor prolamins may be an important gene target in rice breeding, and may reflect cooking properties of rice.

### Conclusion

**Figure 7:**
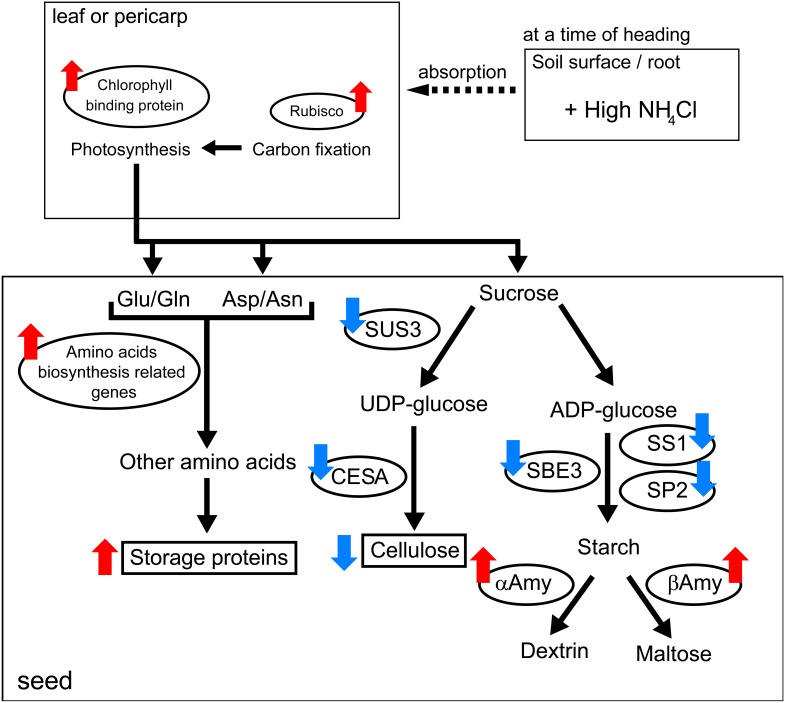
Changes in gene expression and rice seed compounds with additional fertilization. Notable change in mRNA expression and seed compounds are summarized. Molecules in the open oval are DEGs from DNA microarray experiments. Rectangles show the compound whose content was measured in this study. Red and blue arrows indicate up- and down-regulation by additional nitrogen fertilization, respectively. SUS3, sucrose synthase 3; CESA, cellulose synthase A catalytic subunit; SBE3, starch-branching enzyme 3; SS1, starch synthase 1; SP2, starch phosphorylase 2; αAmy, α-amylase; βAmy, β-amylase.

The present data reveal rapid changes in C/N balance in response to rhizosphere nitrogen fertilization at heading time. Notable changes in mRNA expression and seed compounds are summarized in Figure 7. Leaf color showed a darker green and the mRNA expression of Rubisco and chlorophyll-binding protein in seed pericarp was greater in the N-fertilized plot. In general, such situation contributes to increased total photosynthesis, carbon fixation, and nitrogen absorption in the rice plant body, and can improve final yield of grains per unit area [10]. More amounts of sucrose and glutamine/glutamate and asparagine/aspartic acid may be transported into seeds in N-fertilized plot. However, compounds of seeds were not always increased in the N-fertilized plot. The accumulation of nitrogen compounds such as storage proteins increased, whereas that of polysaccharides such as cellulose decreased, correlating with changes in mRNA expression for synthetic processes of these compounds. The mRNA expression profile for starch biosynthesis and starch degradation suggested that levels of other polysaccharides such as starch may also change. The possible mechanism underlying our result is that the amount of carbon backbones may be a limiting factor in seed and it may be primarily used for nitrogen accumulation in endosperm under high nitrogen fertilization (Fig. 7). This study provides new insights into the relationship between fertilization and grain maturation and contributes to the understanding of nutrient distribution during rice production.

## Supporting Information

Figure S1 **Rice of 10 days after additional nitrogen fertilization.**
(PDF)

Figure S2 **Heat map of differentially expressed genes in additional nitrogen fertilization.** Each column represents results from an independent samples. C1, C2 and C3 indicate control samples, and N1, N2 and N3 N-fertilized ones. Each line corresponds to a single probe. The heat map was prepared by obtaining the Z scores from the signal value of each probe after DFW normalization. More reddish and more greenish stand for higher and lower expression levels than the mean, relatively. Significantly enriched Gene ontology (GO) terms (*P*<0.05) and storage substances related genes are shown on the right side of the heat map.
(PPTX)

Table S1 The amino acid content and the protein content of mature polished rice cultivated in a field.
(XLSX)

Table S2 The gene in which expression significantly down-regulated with the additional fertilizer (FDR 0.05>).
(XLSX)

Table S3 The gene in which expression significantly up-regulated with the additional fertilizer (FDR 0.05>).
(XLSX)
