# Supplementary figures and images for: Additional Nitrogen Fertilization at Heading Time of Rice Down-Regulates Cellulose Synthesis in Seed Endosperm
Source: PLoS One. 2014 Jun 6;9(6):e98738. doi: 10.1371/journal.pone.0098738 (PMC4048278; doi:10.1371/journal.pone.0098738)

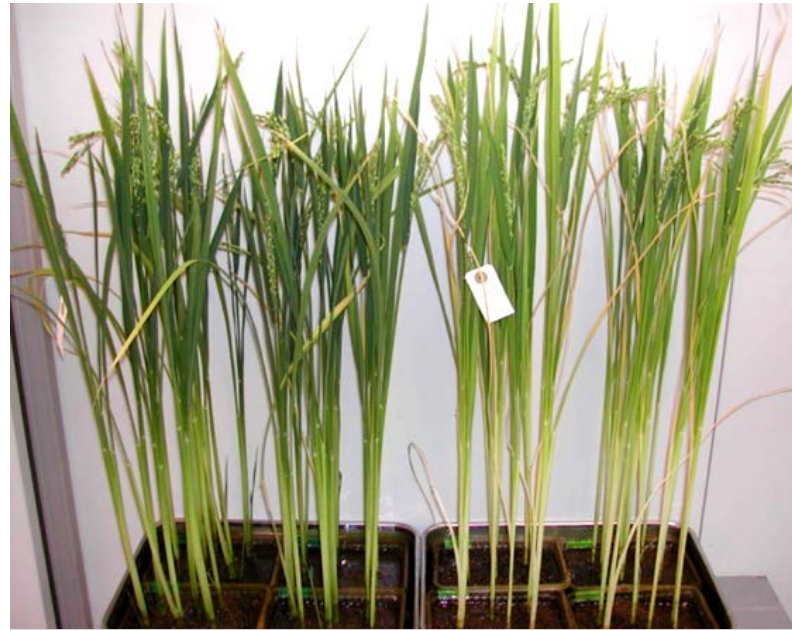

Add  $\text{NH}_4\text{Cl}$

Control

**Figure S1. Rice of 10 days after additional nitrogen fertilization.**

Supplement: Figure S1 — Rice of 10 days after additional nitrogen fertilization. (PDF) [file pone.0098738.s001.pdf]
